# Intracranial aneurysm and arachnoid cyst: just a coincidence? A case report

**DOI:** 10.1590/1516-3180.2017.0083290517

**Published:** 2017-12-18

**Authors:** Guilherme Brasileiro de Aguiar, Rafael Gomes dos Santos, Aline Lariessy Campos Paiva, João Miguel de Almeida Silva, Rafael Carlos da Silva, José Carlos Esteves Veiga

**Affiliations:** I MD, MSc. Attending Neurosurgeon, Faculdade de Ciências Médicas da Santa Casa de São Paulo (FMSCSP), São Paulo (SP), Brazil.; II Medical Student, Faculdade de Medicina da Santa Casa de São Paulo (FMSCSP), São Paulo (SP), Brazil.; III MD. Neurosurgery Resident, Faculdade de Ciências Médicas da Santa Casa de São Paulo (FMSCSP), São Paulo (SP), Brazil.; IV MD. Neurosurgeon, Faculdade de Ciências Médicas da Santa Casa de São Paulo (FMSCSP), and Endovascular Neurosurgery Fellow, Hospital das Clínicas, Faculdade de Medicina da Universidade de São Paulo (HC-FMUSP), São Paulo (SP), Brazil.; V MD. Neurosurgeon, Faculdade de Ciências Médicas da Santa Casa de São Paulo (FMSCSP), São Paulo (SP), Brazil.; VI MD, PhD. Full Professor and Head of Discipline of Neurosurgery, Faculdade de Ciências Médicas da Santa Casa de São Paulo (FMSCSP), São Paulo (SP), Brazil.

**Keywords:** Arachnoid cysts, Intracranial aneurysm, Collagen diseases, Intracranial hemorrhages

## Abstract

**CONTEXT:**

Presence of an arachnoid cyst and a non-ruptured intracystic brain aneurysm is extremely rare. The aim of this paper was to describe a case of a patient with an arachnoid cyst and a non-ruptured aneurysm inside it. Clinical, surgical and radiological data were analyzed and the literature was reviewed.

**CASE REPORT:**

A patient complained of chronic headache. She was diagnosed as having a temporal arachnoid cyst and a non-ruptured middle cerebral artery aneurysm inside it. Surgery was performed to clip the aneurysm and fenestrate the cyst.

**CONCLUSIONS:**

This report raises awareness about the importance of intracranial vascular investigation in patients with arachnoid cysts and brain hemorrhage.

## INTRODUCTION

Intracranial arachnoid cysts account for 1% of all brain lesions.^1^ Most of them are located in the middle fossa.^1^ Because neuroimaging has become more available, arachnoid cysts are becoming diagnosed more frequently, even when they are asymptomatic. This type is the most common presentation,^2^ and constitutes an incidental finding. Patients with arachnoid cysts can also present with headache, nausea and vomiting, and with cranial nerve palsy.^1^ These cysts are acquired lesions relating to abnormal splitting of subarachnoid layers, and they may reach huge dimension. In addition, they may be related to collagen disorder diseases.^1^

On the other hand, the etiology of brain aneurysms is a controversial topic in which genetic changes, smoking and arterial hypertension constitute predisposing factors.^1^ The estimated overall prevalence of unruptured intracranial aneurysms in adults without comorbidities is about 3.2%.^3^ Therefore, although they are common lesions that are often associated with collagen diseases such as Marfan syndrome and polycystic renal disease,^3^ an association between an arachnoid cyst and brain aneurysm in the same patient is extremely rare.^4^^,^^5^

The aim of this paper was to report a case of a patient with a diagnosis of an arachnoid cyst and a non-ruptured intracystic brain aneurysm. The literature on this rare condition was also reviewed.

## CASE REPORT

A 54-year-old female patient presented with a clinical complaint of a left chronic hemicranial headache with pulsatile pattern. In her past medical history, she had only had arterial blood hypertension. Her neurological examination was normal. She did not have any relevant family history.

**Figure 1:**
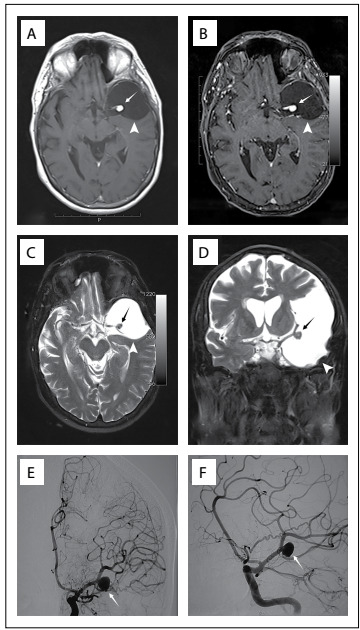
A) Axial T1-weighted non-contrasted magnetic resonance imaging (MRI) showing hypointense lesion at the middle fossa and nodule formation inside the cyst; B) Axial T1-weighted MRI showing hypointense lesion at the middle fossa with saccular dilation inside; C) Axial T2 MRI showing the cyst and the middle cerebral artery bifurcation with saccular dilation at this location; D) Coronal T2 MRI showing the temporal cyst and middle cerebral artery bifurcation with dilation; E) Cerebral arteriography showing aneurysm at the left middle cerebral artery bifurcation; F) Oblique-incidence cerebral angiography showing the aneurysm.

Neurological investigation was performed through brain magnetic resonance imaging (MRI), which revealed a left temporal arachnoid cyst (Figure 1). There were no signs of intracranial bleeding. In addition, localized vascular dilatation at the left middle cerebral artery bifurcation (inside the cyst) was noticed, which was suggestive of saccular aneurysm. Because of this, cerebral angiography was performed, which confirmed the presence of a left middle cerebral artery aneurysm with dimensions of 9 mm x 6 mm and a neck of 3 mm (Figure 1).

**Figure 2:**
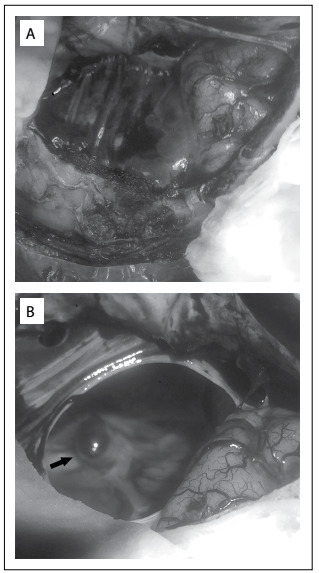
Microsurgical views: A) Before cyst wall opening; B) After cyst wall opening showing the aneurysm at the left middle cerebral artery bifurcation inside it.

Surgical treatment was proposed, consisting of left pterional craniotomy to clip the middle cerebral artery aneurysm. This procedure was implemented without complications. Fenestration of the cyst was also performed to provide communication with the basal cisternae (Figure 2). Only a single clip was needed to achieve occlusion of the aneurysm. The patient presented good recovery, with complete exclusion of the aneurysm from the brain circulation and cyst volume reduction. She presented without neurological deficits and was discharged from hospital for ambulatory follow-up.

## DISCUSSION

Arachnoid cysts are congenital lesions that may cause major neurological symptoms,^1^ but which generally constitute an incidental finding. Because MRI and computed tomography (CT) have become more available, these cysts are becoming diagnosed more frequently. Regarding etiology, there are three main theories:

1. Embryonic dysgenesis during arachnoid cyst formation due to a primary defect of the mesenchyme adjacent to the neural tube;
2. Localized brain agenesis, atrophy or hypoplasia causing secondary expansion of the space for cerebrospinal fluid (CSF);
3. Localized disorder secondary to an inflammatory, infectious, traumatic or hemorrhagic lesion.^1^

Most authors have maintained that arachnoid cysts are congenital, and this theory has been accepted because of their association with other malformations such as corpus callosum agenesis, Marfan syndrome, type 1 neurofibromatosis and polycystic kidney disease.^1^

Similarly, the etiology of saccular brain aneurysms is not well established.^4^ There is an association between collagen diseases and other forms of brain dysplasia, such as in Elhers-Danlos and Marfan syndromes and in polycystic kidney disease.^1^

The current prevalence of brain aneurysms in patients with polycystic kidney disease ranges from 4 to 12%, which is higher than in the general population (1-4%).^3^ In these cases, the risk of rupture risk is higher: about five times greater than in patients without this disease.^3^

Romão et al.^3^ evaluated 92 patients with polycystic disease and found that six of them had some form of intracranial lesion: three with aneurysms and three with arachnoid cysts. However, none of them had both lesions.^3^ It is possible that arachnoid cysts and brain aneurysms are distinct disorders relating to a single dysembryogenesis.^2^ Thus, it can be seen that an association between brain aneurysm and an arachnoid cyst, as in the case reported here (Figures 1 and 2), is very rare.

Arachnoid cysts of the middle fossa only rarely induce neurological symptoms.^5^ These symptoms occur when there is increased pressure on the neighboring structures.^5^ Neurological signs and symptoms may originate from bleeding inside the cyst. Intracystic hemorrhages are generally due to traumatic brain injury (TBI).^1^ Even mild TBI can cause subdural hematomas or intracystic hemorrhage.^1^^,^^5^

Intracystic hemorrhage due to ruptured brain aneurysm is an extremely rare condition,^1^ with few cases reported in the literature. In most cases, the aneurysm is attached to the cyst wall and its rupture gives rise to arachnoid membrane permeation, thus causing intracystic bleeding.^1^ Intracystic hemorrhage is usually caused by rupture of aneurysms of posterior communicating arteries, internal carotid or middle cerebral bifurcations and anterior communicating arteries. These aneurysms may be adjacent to the cyst^1^ and may evolve with intracystic or subarachnoid hemorrhage and subdural hematomas.^2^

An association between brain aneurysm and an arachnoid cyst is a very rare condition (Table 1). A review of the literature was performed through PubMed, searching for the terms “arachnoid cyst” and “intracranial aneurysm” and the few cases reported in the literature were found to describe patients with brain hemorrhage. Therefore, simultaneous arachnoid cyst and non-ruptured brain aneurysm is an even rarer situation.^2^ In the present report, this diagnosis was an incidental finding. de Oliveira et al.^5^ found associations between aneurysms and arachnoid cysts through a review of the literature in which only 10 cases were reported. In most of these cases, intracranial hemorrhage was the first manifestation.^5^

**Table 1:** Search of the literature in medical databases for case reports on intracranial aneurysm and arachnoid cysts

Database	Search strategies	Papers found	Papers related (with ruptured aneurysm)	Papers related (with non-ruptured aneurysm)	Female	Male	Main neurological symptom
MEDLINE (via PubMed, on March 5, 2017)	“Arachnoid cysts” [MESH] AND “Intracranial aneurysm” [MESH] AND Case Reports[ptyp]	23	8	0	6	2	Headache
LILACS (via BVS, on June 1, 2017)	Arachnoid cysts [Palavras] and Intracranial aneurysm [Palavras]	3	2	0	1	1	Headache

There has also been one report of multiple aneurysms associated with arachnoid cysts,^2^ which evolved with rupture of the intracystic aneurysm, without typical subarachnoid hemorrhage.^1^ All of these possibilities should be borne in mind during neuroimaging evaluations on patients with arachnoid cysts without symptoms and on those who present with intracranial hemorrhage.

## CONCLUSION

There is no strong evidence in literature to correlate arachnoid cysts and brain aneurysms. However, for all patients with diagnoses of arachnoid cyst and brain hemorrhage, intracranial vascular investigation should be performed, because these conditions may be associated due to their common pathogenesis. The present unique case of non-ruptured brain aneurysm and arachnoid cyst also serves to raise awareness about the importance of proper vascular investigation, even in cases without intracranial hemorrhage.
